# Intracranial Hemorrhage as a Life-Threatening Complication of Congenital Factor VII Deficiency Associated With Inhibitor Development: A Case Report

**DOI:** 10.7759/cureus.114964

**Published:** 2026-08-22

**Authors:** Sami H Albattat, Hussain A Al Ghadeer, Abbas I Alabdullatif, Ahmed A Bu Amir, Eman F Alamer, Fatimah R Al Hejji

**Affiliations:** 1 Pediatrics, Maternity and Children Hospital, Al Ahsa, SAU; 2 Pediatrics, Imam Abdulrahman Bin Faisal University, Dammam, SAU; 3 Pediatrics, Almaarefa University, Riyadh, SAU

**Keywords:** al ahsa, factor vii deficiency, inhibitor, intracranial hemorrhage, pediatrics, rare bleeding disorders, rfviia, saudi arabia

## Abstract

Congenital factor VII (FVII) deficiency is an uncommon autosomal recessive bleeding disorder with highly variable clinical expression. Although most patients present with mild to moderate bleeding symptoms, severe cases may manifest with life-threatening hemorrhage, particularly intracranial bleeding. The emergence of inhibitors against exogenous FVII is exceedingly rare but poses a significant therapeutic challenge because of treatment refractoriness and an increased risk of major bleeding. We describe a five-year-old Saudi girl with severe congenital FVII deficiency diagnosed in the neonatal period who developed inhibitory antibodies while on regular recombinant activated FVII (rFVIIa) prophylaxis. She presented with acute intracranial hemorrhage and was successfully managed with rFVIIa despite the presence of a high-titer inhibitor. This report highlights the importance of early recognition of inhibitor development in FVII deficiency and the need for individualized therapeutic approaches in rare bleeding disorders.

## Introduction

Rare coagulation factor deficiencies (RCFDs) are a heterogeneous group of bleeding disorders with an estimated incidence of one per 500,000 to one per 2,000,000 persons and account for 5% of total bleeding disorders [1]. Factor VII (FVII) is considered a vitamin K-dependent clotting factor that attaches to tissue factor to activate the extrinsic pathway of the coagulation cascade [2,3]. Deficiency of FVII is one of the RCFDs that is inherited as an autosomal recessive disorder with high prevalence in countries with consanguineous marriages. The severity of FVII deficiency is classified according to the FVII coagulation activity into mild (FVII: C>20%), moderate (FVII: C 10-20%), or severe (FVII: C<10%) [4]. The most commonly presenting symptoms of FVII deficiency are mild to moderate bleeding tendency, including epistaxis, hematoma, menorrhagia, postoperative bleeding, and life-threatening episodes involving the central nervous system, such as intracranial hemorrhage and gastrointestinal bleeding [5]. Usually, it is diagnosed during infancy, and therapeutic options are fresh frozen plasma (FFP), cryoprecipitate, prothrombin complex concentrate (PCC), and recombinant activated FVII (rFVIIa). At the present time, rFVIIa is the mainstay of treatment for FVII deficiency [6]. The development of an FVII inhibitor as a very rare phenomenon accounts for one of the main challenges in the treatment of the disorder. These antibodies cause increased clearance of FVII or neutralize FVII activity [7]. The present study aimed to evaluate the presence of inhibitors against FVII in patients on on-demand or regular prophylaxis treatment with rFVIIa.

## Case presentation

A five-year-old Saudi female with severe congenital FVII deficiency, diagnosed during the neonatal period, was on regular follow-up in the hematology clinic and maintained on prophylactic rFVIIa (NovoSeven) three times weekly. She presented to the emergency department with a three-day history of progressively decreased activity, accompanied by a single episode of nonprojectile, gastric-content vomiting. There was no history of trauma, external bleeding, headache, visual disturbance, abnormal movements, confusion, fever, joint pain, or urinary or skin changes.

Her past medical history was notable for a seizure disorder secondary to a prior intracranial hemorrhage, for which she was maintained on carbamazepine with regular neurology follow-up. She was born at 39 weeks by cesarean section to a 27-year-old primigravida mother following an unremarkable perinatal course. She is the first child of consanguineous parents; the father is a sickle cell trait carrier. There was no family history of bleeding disorders.

At 10 days of life, she developed bleeding from the umbilical stump and was subsequently diagnosed with severe congenital FVII deficiency, with a FVII activity (FVII: C) level of 2% (reference range: 50-150%). Other causes of acquired FVII deficiency (liver disease and vitamin K deficiency) were excluded. At 45 days of age, she experienced her first intracranial hemorrhage, localized to the left parietal lobe, resulting in focal seizures and residual right-sided neurological deficits, including weakness, spasticity, hyperreflexia, and mild facial weakness.

Upon current presentation, the patient was conscious and alert with stable vital signs, though she appeared pale and hypoactive. Neurological examination revealed persistent right-sided weakness (muscle power 4/5 in both the upper and lower limbs), consistent with sequelae of her previous hemorrhage. No new focal neurological deficits were detected. The remainder of the systemic examination was unremarkable. Laboratory findings on admission are summarized in Table 1.

**Table 1:** Laboratory findings on admission with corresponding reference ranges.

Laboratory Test	Result	Reference Range
Hemoglobin	12 g/dL	11.5-15.5 g/dL
White Blood Cell Count	8,000/mm³	4,000-11,000/mm³
Platelet Count	426,000/mm³	150,000-450,000/mm³
Prothrombin Time (PT)	61 seconds	11-15 seconds
International Normalized Ratio (INR)	4.4	0.8-1.2
Activated Partial Thromboplastin Time (aPTT)	38 seconds	25-35 seconds
Factor VII Activity (FVII: C)	2%	50-150%
Factor VII Inhibitor (Bethesda Assay)	7.4 BU	<0.6 BU

**Figure 1:**
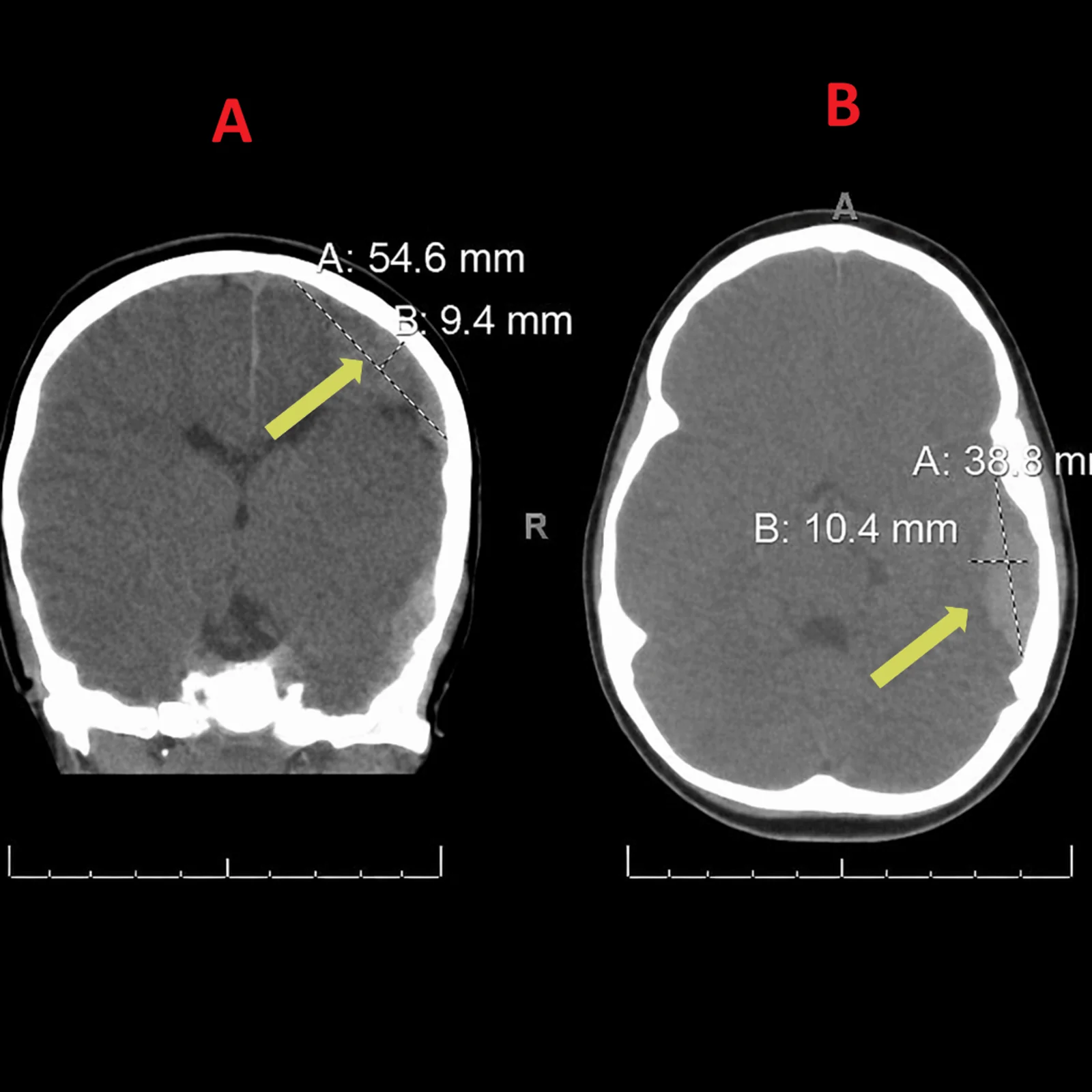
Noncontrast CT images of the brain demonstrating left-sided extra-axial hemorrhage. (A) Coronal CT image demonstrating a left high parietal subdural hematoma measuring approximately 5.4×0.9 cm, with mixed densities suggestive of a subacute component (arrow). An underlying left parietal intraparenchymal hemorrhagic focus measuring approximately 18×18 mm is also noted. (B) Axial CT image showing a left temporal acute epidural hematoma with a lentiform configuration measuring approximately 3.8×1.0 cm (arrow). The ventricular system is normal in size and configuration, with no evidence of midline shift or significant mass effect. The midbrain and posterior fossa structures appear unremarkable.

**Figure 2:**
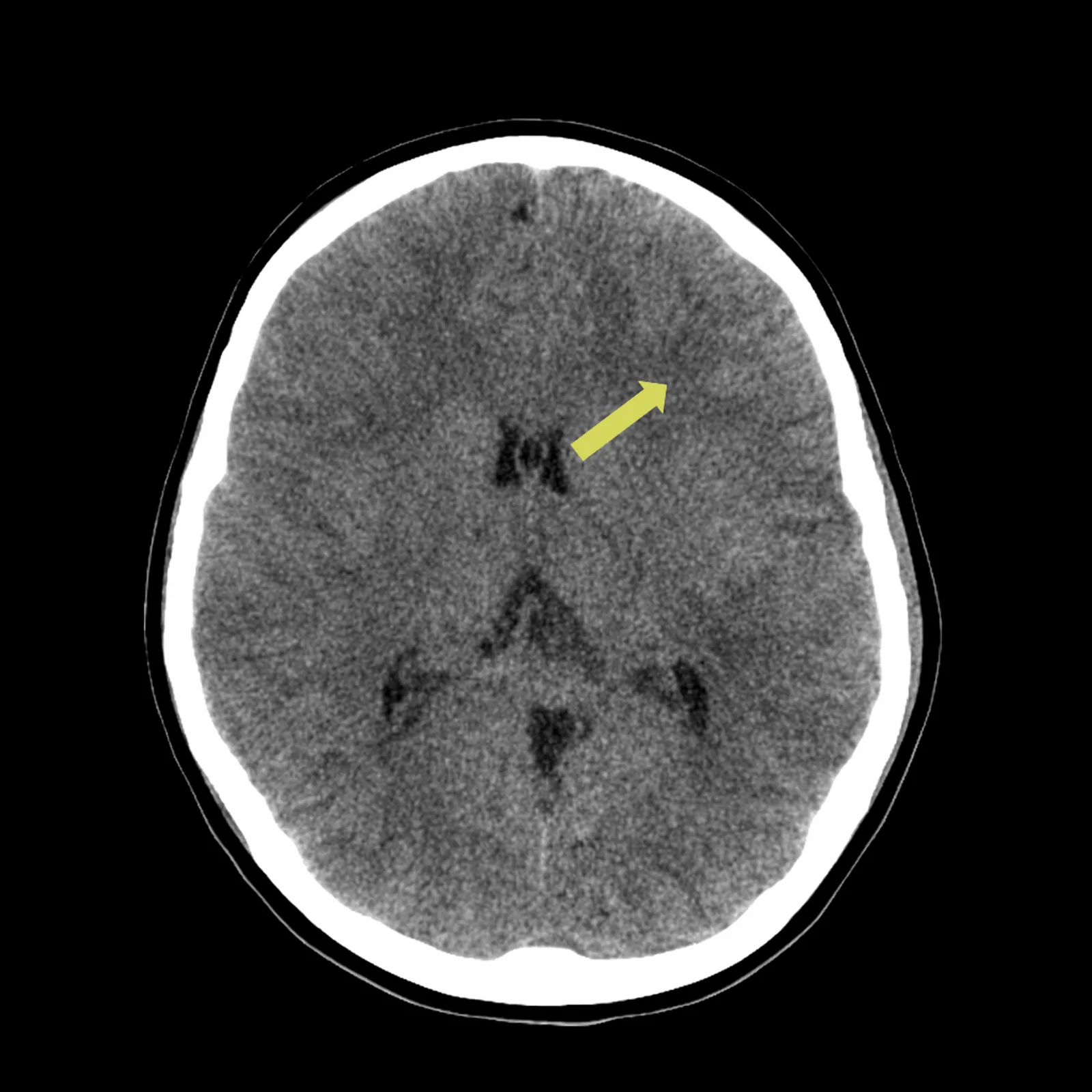
Axial noncontrast CT image with enhanced resolution illustrating cortical-subcortical involvement and subtle internal hyperdensities. This figure demonstrates a left high parietal cortical-subcortical wedge-shaped area with subtle linear hyperdensities within it (arrow). No other areas of acute hemorrhagic blood products are identified. No additional abnormal areas of increased or decreased attenuation are detected. The ventricular system is normal in size and configuration. There is no evidence of intra-axial or extra-axial collections or space-occupying lesions. No mass effect or midline shift is observed. The midbrain and posterior fossa structures appear unremarkable.

**Figure 3:**
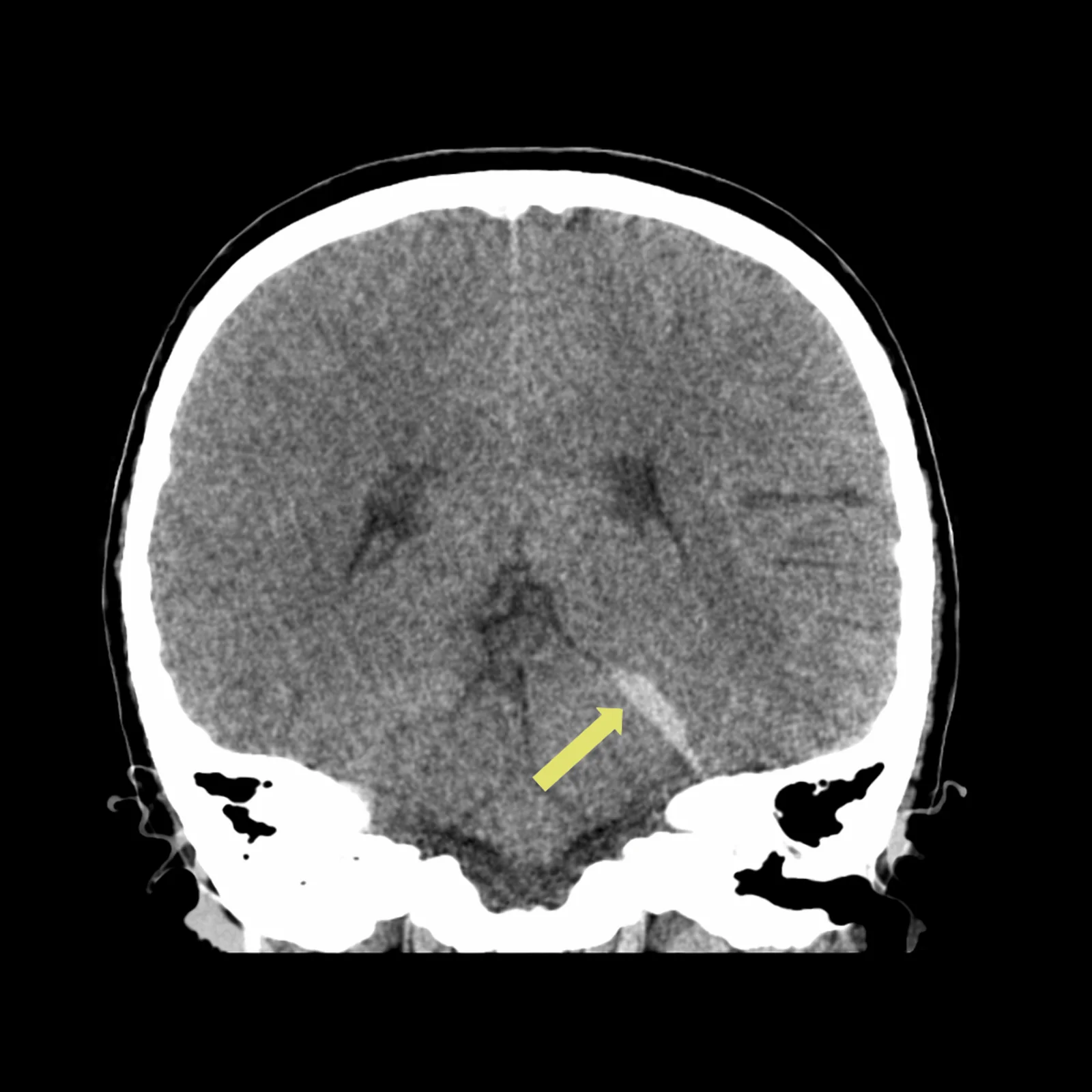
Coronal noncontrast CT image showing the same left high parietal cortical-subcortical wedge-shaped abnormality. Noncontrast CT of the brain demonstrates a left high parietal cortical-subcortical wedge-shaped area containing subtle linear hyperdensities (arrow). No other areas of acute hemorrhagic blood products are identified. There are no additional abnormal areas of increased or decreased attenuation. The ventricular system is normal in size and configuration. No intra-axial or extra-axial collections or space-occupying lesions are seen. There is no evidence of mass effect or midline shift. The midbrain and posterior fossa structures appear unremarkable.

Initial non-contrast CT of the brain demonstrated multiple intracranial hemorrhages, including a left temporal epidural hematoma and a left high parietal subdural hematoma with mixed attenuation compatible with a subacute stage, in addition to a small underlying intraparenchymal hematoma (Figure 1). Follow-up CT scans at two weeks (Figure 2) and one month (Figure 3) demonstrated resolution of acute hemorrhage with a persistent left high parietal cortical-subcortical wedge-shaped hypodensity, consistent with an evolving posthemorrhagic infarction. No midline shift, mass effect, ventricular abnormality, or posterior fossa involvement was noted across all studies.

On admission, rFVIIa (NovoSeven) was administered according to established treatment protocols for acute bleeding in congenital FVII deficiency [5]. An initial dose of 90 µg/kg was given intravenously and repeated every two to three hours during the acute phase until clinical stabilization was achieved. Following improvement in the patient’s neurological status and radiologic stabilization of the hemorrhage, the dosing interval was gradually extended. The patient was subsequently maintained on regular prophylaxis with rFVIIa, 30 µg/kg administered two to three times weekly, to reduce the risk of recurrent bleeding. Supportive management and close neurological and hematologic monitoring were also performed during hospitalization. A repeat FVII assay remained at 2%. Screening for inhibitors using an adapted Bethesda assay demonstrated an inhibitor titer of 7.4 Bethesda Units (BU) (reference: <0.6 BU considered negative) after a two-hour incubation at 37°C, confirming the development of FVII inhibitors.

The patient’s intracranial hemorrhage gradually resolved on follow-up imaging. She continued on rFVIIa prophylaxis and has demonstrated good clinical stability. She is now five years old, stable, neurologically improving, off antiepileptic medications, and maintained on routine outpatient hematology follow-up with no further major bleeding events.

Clinical timeline

The patient was diagnosed with congenital FVII deficiency during early infancy and was started on prophylactic rFVIIa therapy. She remained clinically stable with intermittent follow-up until the age of five years, when she presented with an acute intracranial hemorrhage. During the evaluation of this bleeding episode, laboratory testing revealed the presence of a FVII inhibitor with a Bethesda titer of 7.4 BU. The patient was treated with rFVIIa, resulting in successful control of the bleeding episode, and was subsequently maintained on prophylactic therapy with close hematologic follow-up.

## Discussion

Hemostasis is a complex physiological process that prevents excessive bleeding following vascular injury while maintaining normal blood flow. It involves coordinated interactions between the vascular endothelium, platelets, and coagulation factors that promote clot formation at the site of injury [1]. Defects in coagulation factors may result in bleeding disorders of varying severity. RCFDs are a heterogeneous group of inherited disorders characterized by quantitative or qualitative defects in coagulation proteins, accounting for approximately 3-5% of all inherited bleeding disorders. Clinical manifestations range from mild mucocutaneous bleeding to life-threatening hemorrhage [8,9]. Among RCFDs, congenital FVII deficiency is the most frequently reported disorder, with an estimated prevalence of approximately one in 500,000 individuals worldwide [8-10]. First described by Alexander et al. in 1951, the condition follows an autosomal recessive inheritance pattern and results from mutations in the F7 gene located on chromosome 13, which encodes the vitamin K-dependent coagulation FVII protein [11,12]. More than 250 pathogenic variants have been identified, most of which are missense mutations, while splice-site mutations, nonsense mutations, and small deletions have also been reported [13]. A distinctive feature of congenital FVII deficiency is the poor correlation between plasma FVII activity levels and clinical bleeding severity. Approximately one-third of patients remain asymptomatic, whereas two-thirds experience mild to moderate bleeding symptoms, and only 10-15% develop severe hemorrhagic manifestations [12,14]. The most serious complications include gastrointestinal and central nervous system (CNS) hemorrhage, particularly in infancy and early childhood, accounting for up to 60-70% of severe bleeding episodes in this age group [15]. The disorder is generally classified according to plasma FVII activity levels as mild (FVII: C>20%), moderate (FVII: C 10-20%), or severe (FVII: C<10%) [4]. In the present case, the patient was diagnosed with severe congenital FVII deficiency during the neonatal period following umbilical bleeding and subsequent intracranial hemorrhage. The presence of parental consanguinity may have contributed to the occurrence of this rare autosomal recessive disorder.

Management primarily involves replacement therapy aimed at restoring hemostatic activity during bleeding episodes or as prophylaxis in severe cases [6,9]. Therapeutic options include rFVIIa, PCC, plasma-derived FVII concentrates, and FFP. In selected severe cases with recurrent bleeding and poor response to therapy, liver transplantation has also been reported as a potential curative option [4,16]. rFVIIa is currently considered the preferred treatment due to its efficacy and favorable safety profile. Despite its effectiveness, replacement therapy carries a risk of alloantibody formation against the administered coagulation factor, which may reduce therapeutic efficacy and increase the risk of bleeding complications [4,16]. However, inhibitor development in congenital FVII deficiency is extremely rare compared with other coagulation disorders such as hemophilia A and B [3,17]. In our case, the patient had been receiving prophylactic rFVIIa (NovoSeven) since early infancy. At five years of age, she developed an inhibitor that was detected following an unexpected bleeding episode and reduced response to replacement therapy. Laboratory evaluation confirmed the presence of an inhibitor with a Bethesda titer of 7.4 BU. Despite the presence of this inhibitor, bleeding control during the acute episode was successfully achieved with continued administration of rFVIIa. This may be explained by the pharmacologic effect of rFVIIa at supraphysiologic concentrations, which can partially overcome inhibitory activity and promote thrombin generation. No change in the therapeutic agent was required, although close clinical and hematologic monitoring was maintained.

Previous studies have reported variable incidence rates of inhibitor development in congenital FVII deficiency. A systematic review of 13 studies published between 1990 and 2020 identified inhibitors in 27 of 380 patients, corresponding to an incidence of approximately 7%. Data from the international Seven Treatment Evaluation Registry (STER) reported an incidence of 2.6% (three out of 115 patients) [16]. Similarly, studies from Iran reported incidence rates of 4% and 8.8%, demonstrating variability across different populations and registries [18]. Regional epidemiological studies also demonstrate differences in the distribution of rare bleeding disorders. In Korea, only 20 patients with congenital FVII deficiency without inhibitors have been reported [19]. In Saudi Arabia, a retrospective study evaluating rare bleeding disorders found that FVII deficiency accounted for approximately 15.4% of cases (six out of 39 patients), with a strong association with parental consanguinity and positive family history [20]. Patients who develop inhibitors are at significantly higher risk of severe bleeding complications, particularly intracranial hemorrhage. This complication has been reported in approximately 60% of patients with inhibitors compared with about 10% among those without inhibitors [21]. Other commonly reported manifestations include epistaxis, gastrointestinal bleeding, and soft-tissue hematomas. A systematic review of 27 patients with congenital FVII deficiency and inhibitors reported intracranial hemorrhage in 58% of cases, followed by epistaxis (46%), hematoma (35%), and gastrointestinal bleeding (31%) [8,22].

Our patient’s clinical course is consistent with these findings, as she developed an inhibitor (7.4 BU) and presented with intracranial hemorrhage at the time of inhibitor detection. This case is notable because an intracranial hemorrhage occurred concurrently with the identification of the inhibitor, which is an uncommon and potentially life-threatening presentation in congenital FVII deficiency. In addition, despite the presence of the inhibitor, bleeding control was successfully achieved using rFVIIa without the need to change the therapeutic agent. Management of bleeding in such patients remains challenging due to the limited number of reported cases and the absence of standardized treatment guidelines. rFVIIa may remain effective in some cases when administered at higher or repeated doses, although treatment response may vary [8,18]. Therefore, individualized treatment strategies and further data from international registries are needed to optimize management in this rare condition.

## Conclusions

Congenital FVII deficiency is a rare bleeding disorder with highly variable clinical manifestations. The development of inhibitors against rFVIIa is an extremely uncommon but serious complication that may significantly increase the risk of life-threatening hemorrhage, including intracranial bleeding. Early recognition of inhibitor formation is essential, particularly in patients who present with unexpected bleeding episodes or a reduced response to replacement therapy.

This case highlights the importance of careful clinical monitoring and individualized management strategies in patients with severe FVII deficiency receiving long-term replacement therapy. Increased awareness of this rare complication may facilitate timely diagnosis and appropriate therapeutic interventions, ultimately improving patient outcomes.
